# Estimation of the Kinetic Coefficient of Friction of Asphalt Pavements Using the Top Topography Surface Roughness Power Spectrum

**DOI:** 10.3390/ma18153643

**Published:** 2025-08-02

**Authors:** Bo Sun, Haoyuan Luo, Yibo Rong, Yanqin Yang

**Affiliations:** 1 Bowen Construction Co. Ltd., Kunming 650217, China; sunbokust@163.com (B.S.);; 2 Faculty of Transportation Engineering, Kunming University of Science and Technology, Kunming 650500, China

**Keywords:** asphalt pavement, surface texture, rubber friction, top topography, kinetic coefficient of friction, power spectrum

## Abstract

This study proposes a method for estimating the kinetic coefficient of friction (COF) for asphalt pavements by improving and applying Persson’s friction theory. The method utilizes the power spectral density (PSD) of the top surface topography instead of the full PSD to better reflect the actual contact conditions. This approach avoids including deeper roughness components that do not contribute to real rubber–pavement contact due to surface skewness. The key aspect of the method is determining an appropriate cutting plane to isolate the top surface. Four cutting strategies were evaluated. Results show that the cutting plane defined at 0.5 times the root mean square (RMS) height exhibits the highest robustness across all pavement types, with the estimated COF closely matching the measured values for all four tested surfaces. This study presents an improved method for estimating the kinetic coefficient of friction (COF) of asphalt pavements by employing the power spectral density (PSD) of the top surface roughness, rather than the total surface profile. This refinement is based on Persson’s friction theory and aims to exclude the influence of deep surface irregularities that do not make actual contact with the rubber interface. The core of the method lies in defining an appropriate cutting plane to isolate the topographical features that contribute most to frictional interactions. Four cutting strategies were investigated. Among them, the cutting plane positioned at 0.5 times the root mean square (RMS) height demonstrated the best overall applicability. COF estimates derived from this method showed strong consistency with experimentally measured values across all four tested asphalt pavement surfaces, indicating its robustness and practical potential.

## 1. Introduction

The safety of an asphalt pavement is related to several aspects, such as drivers, vehicles, pavement surfaces, and environmental conditions [1]. Despite the subjective effects, the surface friction, or skid resistance, is closely connected to the pavement surfaces. Pavement with insufficient surface friction may lead to traffic accidents, especially on rainy days. These accidents are caused by various reasons, including driving mistakes, vehicle defects, and insufficient friction at the tire–pavement interface. The surface characterizations of asphalt pavement are critical for driving safety and comfort. The asphalt pavement should provide enough friction and maintain a certain degree of ride quality to meet the requirements of the driving public. Therefore, the key parameters of designing a surface course consist of good friction, low levels of roughness, and low levels of noise [2].

Friction mainly depends on the contact behavior between the road surface texture and the tread rubber [3]. Increasing the texture on the surface course will improve the pavement’s performance in terms of skid resistance. Modern rubber friction theory argues that adhesion and hysteresis are two main components of the friction between tires and pavements [4]. In case of high traveling speeds and wet conditions, the hysteresis becomes the main contributor to friction. Wear of the micro-texture on the top surface will lead to a significant reduction in hysteresis, resulting in a loss of skid resistance of pavement. Accordingly, many studies developed friction models to evaluate the pavement skid resistance quantitatively derived from the tire–road contact theory. Greenwood and Williamson [5] assumed a rough pavement is a mean plane on which there are a large number of randomly distributed spherical roughs of fixed radius. And they built the Greenwood and Williamson contact theory (G-W) based on the elastic contact as well as the hysteretic friction of rubber on a single spherical asperity described by the classical approach of Hertz [6]. Greenwood and Tabor [7] further suggested that the pressure dependence of the coefficient can be explained in terms of elastic hysteresis, combined in the case of the sharper cones with tearing of the rubber. Klüppel [8] presented the concept of rubber friction on rough surfaces, which relates the frictional force to the dissipated energy of the rubber during sliding stochastic excitations on a broad frequency scale. It is worthwhile to note that in studies of Klüppel, an asphalt road surface was considered as a surface with random fractal roughness so that the self-affine curve can be used to describe them [9,10]. This is recognized by many scholars and is believed to provide a general approach for many subsequent studies using functional relationships to describe pavement profiles. After further optimizing and combining the hysteresis excitation energy dissipation theory of rubber and the self-affine model of the pavement, Persson [11,12] employed the surface roughness power spectrum (PSD) to characterize the randomly rough surfaces in multiscale, and then developed a theory of rubber friction for the sliding of a rubber block on a rough surface with roughness on many different length scales. Persson’s theory was validated in many experimental studies and has also been used to define interaction behaviors in studies involving the analysis of tire–road contact by finite element methods [13,14]. However, rubber friction on road surfaces is a complex problem with different contributory factors. As a result, it is sometimes difficult to find a strong correlation between the coefficients of friction (COF) calculated by Persson’s theory and those measured in the field [15].

The main reasons for the deviation of COF between the theoretical and experimental values include: (I) in Persson’s theory, the contributions of adhesion are ignored, which leads to the deviations especially at low speeds [16]; (II) the temperature of the rubber in friction is always changing, which affects its material properties (modulus and phase angle) [16,17]; and (III) only the top surface of the pavement is actually in contact with the tire, so that it is not necessary for all surface texture data to be involved in the calculation [18].

For (I), Lorenz supplemented Persson’s theory by adding shear processes in the area of contact (it arises from rubber molecules (or patches of rubber) undergoing bonding-stretching-debonding cycles, i.e., the adhesion) as an important contribution to the rubber friction at low speeds. Lorenz also provided a theoretical formula for calculating the adhesion [16]. For (II), Lorenz and Persson [19] offered a possible solution where the friction (shear stress) curves calculated at multiple temperatures were pieced together into a friction master-curve using the time-temperature equivalence principle, i.e., via the WLF equation [16]. For (III), some recent studies have shown that the friction on the pavement surface is significantly more correlated with the top surface roughness than with the overall roughness. Actually, this was also first proposed by Persson [18]. Some other studies investigated the correlation between the top roughness and the friction with rubber. Hartikainen et al. [20] proved this by a field experiment, where the highest correlations were found at evaluation depths of 0.5 times the average root mean square ($S_{q}$) of the total analyzed surfaces. Deng et al. [21] studied the correlation characteristics between top roughness and BPN for three typical dense-graded pavements (AC-10, AC-13, and AC-16), and they found that the greater the roughness of the road surface, the greater the area ratio of the effective top topography to the total topography. Ding et al. [22] and Kogbara et al. [23] derived the effective tire–pavement contact range within the top 2 mm of the surface based on the statistical relationship between the three-dimensional road surface textures and the measured COFs. Du et al. [24] and Mahboob Kanaf et al. [25] yielded results characterized by the area ratio of the topography, where ~20% was their reported effective rubber-contacted range.

Overall, the current published studies all indicate that the friction is better correlated with top topography compared to the total topography. Moreover, the top topography PSD appears to be the best technique to characterize the road surface profiles for tire–pavement contact [20]. However, knowledge gaps remain in understanding the friction behavior subject to pavement top topography characteristics. First, although existing studies have shown that the calculations of the contact mechanics using any top topography PSD other than the top 50% lead to false conclusions [25], the range of top topographic depths of different pavement types involved in friction is unclear. There is disagreement on how to determine the depth of cut for the top texture. More importantly, published works only reported the relationship between topography and COFs at a fixed velocity, but few have investigated the dynamic effects of velocity on this relationship.

We aim to establish an approach for estimating the kinetic COF combined with the top topography surface roughness PSD and Persson’s rubber friction. The core of this is which percentage of the top topography is appropriate to use to estimate the COF. In other words, how to select the cutting plane of the top topography. In this study, four cutting methods, including the 0.5 $S_{q}$ recommended by Hartikainen [20], the fixed area ratio (top 20%) by Mahboob Kanaf [25], the interface separation, and the upper half of the top topography (top 50%) by Persson [18,26] were compared. They were used to estimate the kinetic COF of four typical asphalt pavements (SMA-16, SMA-13, AC-13, and OGFC-5) in the rubber sliding velocity range of 0~80 km/h, and the results were verified by the field measurement of a dynamic friction tester (DFT).

## 2. Persson’s Friction Theory Without Consideration of Top Texture

### 2.1. Persson’s Rubber Friction Theory

Modern rubber friction theory believes that adhesion and hysteresis are the two main elements of the friction when a rubber slider slides on a solid and rough surface [27], as follows:

(1) $${COF}_{total}={COF}_{Adh}+{COF}_{Hys}$$

where ${COF}_{total}$ is the total coefficient of friction between the rubber and pavement, no unit; and ${COF}_{Adh}$ and ${COF}_{Hys}$ are the coefficients of friction for adhesion and hysteresis, respectively, no unit.

Persson demonstrated that the contribution of the adhesion part to the total friction is significantly reduced in cases of contact surfaces contaminated with dust or liquids, and at high driving speeds [11]. Lorenz [16] further proved that the adhesion part only exhibits significance at low speeds, but it can be almost ignored when the speed reaches the regular running speed of the car. Therefore, the contribution of hysteresis is considered the dominant factor in the friction of a tire and a rough surface at high speeds, and the adhesion part is selectively ignored in most published rubber friction models [5,6,7,8,11]. Currently, a well-known theoretical model to calculate the coefficient of friction (${COF}_{total}$) between rubber and rough surface is presented by Persson [11], as follows:

(2) $${COF}_{total}\approx {COF}_{Hys}=\mu_{persson}=\frac{1}{2}\int_{q_{L}}^{q_{1}}dq\;q^{3}C(q)P(q)\int_{0}^{2\pi }d\phi \;cos\phi Im\frac{E(qvcos\phi )}{(1-\upsilon^{2})\sigma_{0}}$$

(3) $$P\left(q\right)=\frac{2}{\pi }\int_{0}^{\infty }e^{-x^{2}G(q)}dx\approx {\left[\pi G(q)\right]}^{-0.5}$$

(4) $$G\left(q\right)=\frac{1}{8}\int_{q_{L}}^{q}dq\;q^{3}C(q)dq\int_{0}^{2\pi }d\phi {\left|\frac{E(qvcos\phi )}{(1-\upsilon^{2})\sigma_{0}}\right|}^{2}$$

where q is the wave vector obtained by the Fourier expansion of the pavement profile, with unit m^−1^;$\;q_{1}=2\pi /\lambda_{1}$ and $q_{L}=2\pi /\lambda_{L}$, respectively, present the upper cutoff and bottom cutoff wave vector at the wavelength of $\lambda_{1}$ and $\lambda_{L}$, with unit m^−1^; $\lambda_{1}$ and $\lambda_{L}$, respectively, present the minimum wavelength and maximum wavelength of the wave vector obtained by the Fourier expansion of the pavement profile, with unit m; υ is the Poisson’s ratio of the rubber, typically ~0.5 [18]; $\sigma_{0}$ is vertical pressure stress to the rubber,~0.15 MPa in DFT; v is the sliding velocity of the rubber, m/s; and the ϕ is the angle between the v and q, °; Im stands for to extract the imaginary part of the complex modulus of the rubber (E); and C(q) is the PSD of the pavement profile, which is essentially a Fourier expansion of the autocorrelation function of the pavement surface amplitude. A typical method to obtain the C(q) is provided by Welch and introduced in Persson’s work [12] as follows:

(5) $$C\left(q\right)=\frac{1}{{\left(2\pi \right)}^{2}}\int d^{2}X\;\langle z\left(X\right)z\left(0\right)\rangle e^{-iq.X}$$

where $z\left(X\right)$ is the asperity amplitude at X=(x, y) measured from the average plane that z=0, which represents the ensemble averaged over the surface area; and e is the natural constant; $\langle \ldots \rangle$ stands for to calculate the ensemble averaging.

It should be noted here that the integration boundaries $q_{1}$ (upper cutoff wavenumber) and $q_{L}$ (bottom cutoff wavenumber) may affect the final results of COF, significantly, but there is no clear definition of how these two values should be taken in previous studies [11]. In the current study, $q_{L}$ is equal to 2π/L. L is the nominal contacting length of contact area between rubber slider and pavement, unit m; $q_{1}$ is equal to $2\pi /\lambda_{1}$. $\lambda_{1}$ is the minimum wave take out from the pavement profile wave by Fourier expansion, unit m. Persson’s and previous [12,28] studies argued that it is economical for calculating a COF with sufficient precision that $\lambda_{1}$ taken to the range of magnitude of ~μm, I. e., 10^−5^ m.

In Equations (2)–(4), E(qvcosϕ) is the complex modulus of the rubber, with unit MPa; and qvcosϕ is the loading frequency (ω) to the rubber. Im means taking the imaginary part of the composite modulus, i.e., the loss modulus of rubber. This parameter requires the data of the viscoelastic modulus master-curve of the rubber.

### 2.2. Top Topography PSD

**Figure 1:**
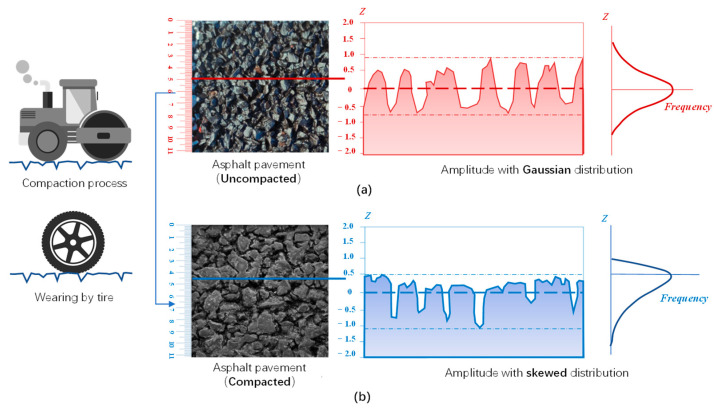
Amplitude distribution of the pavement surface shift from a Gaussian distribution (**a**) to a skewed distribution (**b**) under the effects of compaction and wearing by tires.

**Figure 2:**
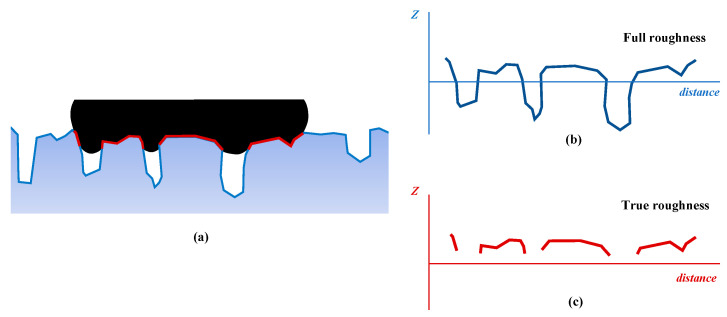
Actual interface between rubber and pavement (**a**), full roughness of the surface (**b**), and the true roughness derived from the actual interface (**c**).

In Section 2.1, the method following Persson’s initial theory for estimating the COF between the rubber and pavement was described. In this process, the C(q) inputted to calculate the COF is derived from the total rough surfaces in multiscale (recall Equation (5)). However, some recent studies [20,24,29] indicated that the friction of a pavement surface had a higher correlation with the C(q) calculated on the top profile than that on calculated total profile. The reason for these is that the amplitude distribution of the pavement surface is not a Gaussian random distribution, but is a skewed due to the compaction process during the construction and the wearing effect during the road service. Effects of the compaction and the wearing by tire tended to reorient the aggregates so that the top surface tend to align with the compaction plane [20], as shown in Figure 1. In this context, the upward peaks on the pavement surface were flattened and lost the random roughness (on the current or smaller scale), but the downward valleys were not affected by these and remain in a more randomly rough state. When the rubber tire contacts the skewed pavement surface, the actually interface only concentrates on the flat top of these aggregates and a small part penetrates into the upper of valleys, as the red area in the shown in Figure 2a. It difficult for making full contact on the surface of bottom of valleys, because the local pressure of rubber must be reach the order of E ≈ 10 MPa to full the valleys in current scale (The vertical pressure of tires caused by a normal heavy-duty truck would not exceed 1 MPa) [11]. Therefore, only the top roughness (the are actually contact with rubber Figure 2c) but not the full roughness (Figure 2b) on the surface makes the actual contribution to the friction between the rubber and pavement. Consequently, some studies proved that it was better correlated with the experimental data of tire–road friction if the top topography PSD ($C_{T}(q)$) is used instead of the total topography PSD (C(q)) in calculating the theorical COF by Persson’s friction theory (Equations (2)–(5)). It should be noted that the discontinuous line segments in Figure 2c only represent the actual contact area at the current scale, while each of them is actually broken at the contact interface as well as at the current scale at a smaller observation scale.

The approach to compute the PSD of the top topography ($C_{T}(q)$) of a pavement was proposed by Persson et al. and improved by Hartikainen et al. as follows:

(6) $$C_{T}(q)=\frac{N}{N_{T}}\cdot \frac{1}{{\left(2\pi \right)}^{2}}\int d^{2}X\langle z_{T}\left(X\right)z_{T}\left(0\right)\rangle e^{-iq.X}$$

where N is the total number of data points in the full surface; $N_{T}$ is the corresponding number of data points in the top topography; and $z_{T}\left(X\right)$ and $z_{B}\left(0\right)$ are the asperity amplitude at positions of X=(x, y) and of X=(0, 0) of the top topography measured from the average plane that z=0. $\langle \ldots \rangle$ stands for to calculate the ensemble averaging.

Similarly, the PSD of bottom topography ($C_{B}(q)$) is calculated as follows:

(7) $$C_{B}(q)=\frac{N}{N_{B}}\cdot \frac{1}{{\left(2\pi \right)}^{2}}\int d^{2}X\langle z_{B}\left(X\right)z_{B}\left(0\right)\rangle e^{-iq.X}$$

where N is the total number of data points in the full surface; $N_{B}$ is the corresponding number of data points in the bottom topography; and $z_{B}\left(X\right)$ and $z_{B}\left(0\right)$ are the valley amplitudes at positions of X=(x, y) and X=(0, 0) of the bottom topography measured from the average plane that z=0. $\langle \ldots \rangle$ stands for to calculate the ensemble averaging.

### 2.3. Optimal Cutting Plane of Top Topography

**Figure 3:**
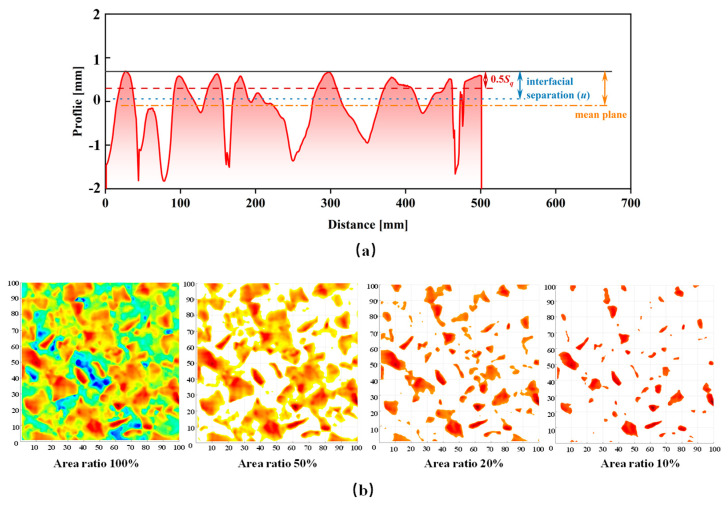
Determining the optimal cutting plane of top topography by depths (Persson’s [18], Hartikainen’s [20], and interfacial separation [26] theory) (**a**) or fixed area ratio (Mahboob Kanaf’s theory [25]) (**b**).

To overcome the negative effects of the skewed height distribution of pavement topography described above, Persson et al. [18] suggested only using the surface data above the mean plane to calculate the PSD, as shown by the yellow line in Figure 3a. In fact, the depth of the cutting plane determines the length scale of the pavement texture involved in the calculation. A deeper cutting plane (a thicker range of the top topography) means that larger scales of texture on the surface are taken into account. Therefore, the location of the cutting plane directly affects the PSD and the final prediction of the pavement friction coefficient. Hartikainen [20] reported a higher correlation ($R^{2}\approx 0.82$) between the top topography PSD and experimental data of friction obtained when the depth of the cutting plane is equal to 0.5 times the root mean square ($S_{q}$) of the surface roughness, as the red line shown in Figure 3a. Another simple method of determining the cutting plane was provided by Mahboob Kanaf et al. [25], in which a fixed ratio of the area of the top topography was directly used, as shown in Figure 3b. A high correlation coefficient with an average $R^{2}>0.8$ was found between the friction and the top 20% (area ratio) of topography in her report. In addition to these methods, Persson presented a theoretical approach to estimate the cutting depth by means of the interfacial separation (u) [26]. Actually, Persson derived a relation between the average interfacial separation and the normal load for the contact of a rigid solid with a randomly rough surface based on elastic deformation theory of rubber, as follows:

(8) $$u\approx \gamma S_{q}log\left(0.5{\epsilon q}_{0}S_{q}E^{\prime }/p\right)$$

where p is the vertical pressure on the rubber; ϵ is a constant value, ≈0.7493; $q_{0}$ is the roll-off wavevector of the surface (in case of rough surface of asphalt pavements; $q_{0}$ represents the nominal maximum aggregate sizes (MNAS) in a pavement roughness PSD); $E^{\prime }$ is the elastic modulus of rubber; and γ ≈ 0.4~0.5 [26,30].

Interfacial separation is determined by both the nature of the rubber and the texture characteristics, which means that it is not a fixed value for the same pavement and changes with the tire (rubber). But the depth of the top topography created by the other methods is fixed. In this study, the four methods were used to determine the cutting plane of the top topography, and the effect on the predicted coefficients of friction was checked.

## 3. Materials and Methods

### 3.1. Pavement Samples

**Figure 4:**
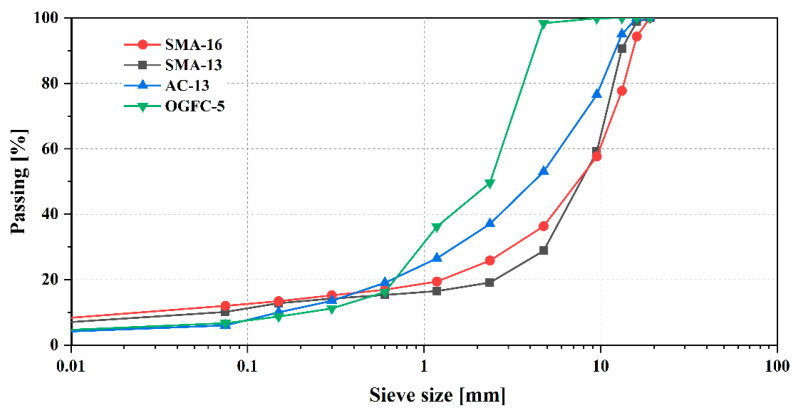
Aggregate gradation curve.

The types of mixture are important in the performance of the asphalt pavement. In this study, four types of asphalt mixtures were selected as the research objects, including two stone matrix asphalt mixtures with the nominal maximum aggregate sizes (NMAS) of 13.2 mm (SMA-13) and 16 mm (SMA-16), an asphalt concrete with a NMAS of 13 mm (AC-13), and an open-grade friction course mixture with a NMAS of 5 mm (OGFC-5). The gradations of the four mixtures are shown in Figure 4. It should be noted that the SMA-16 and SMA-13 mixtures exhibit gap-graded characteristics, while the AC-13 and OGFC-5 mixtures possess dense-graded and open-graded characteristics, respectively.

**Figure 5:**
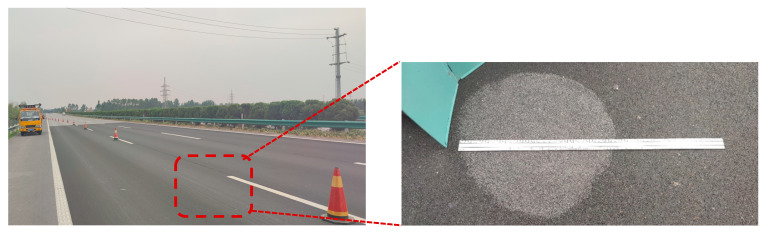
The construction site of the new expressway in Henan Province.

The detail of the mixture design is shown in Table 1. The SMA-16 and SMA-13 mixture used the base binder without any additives. The SMA-13 mixture has 6.5% SBS modified binder and 0.055% fibers. The OGFC-5 mixture has 5.5% high-viscosity modified binder. All of these four pavement samples were directly acquired from a new freeway site in Henan province, China, as shown in Table 1. The on-site photos are shown in Figure 5.

### 3.2. Data Acquisition and Preprocessing

#### 3.2.1. Digitized Pavement Texture by 3D Laser Scanning

**Figure 6:**
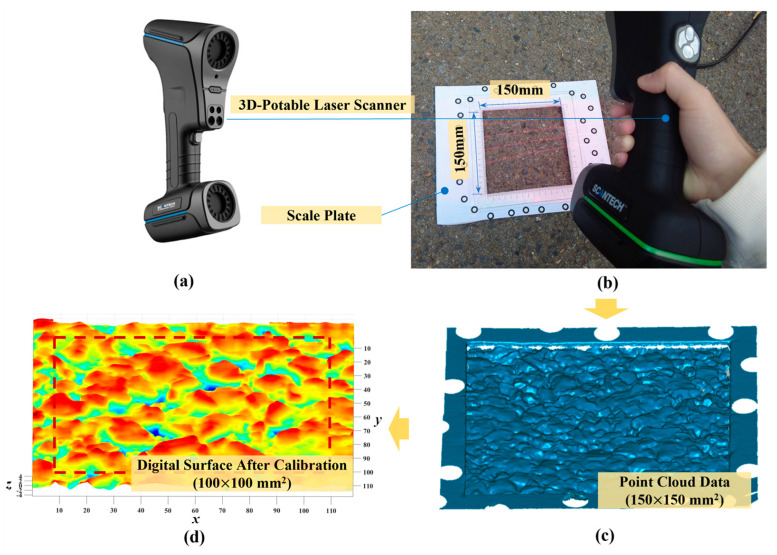
Scanning a surface of pavement sample (**a**) boxed in the special scanning plate (**b**), and calibrating their original point cloud data (**c**) to the effective digital surface (**d**).

In this study, the surface topography data were collected using a KSCAN-20 3D portable laser scanner produced by Hangzhou SCANTECH^®^ Optical Technology Co., LTD of China (Hangzhou, China), as shown in Figure 6a. By sending 14 crossed laser beams and receiving these laser beams from the pre-set reflective marking dots, this laser scanner can export the 3D coordinate information of the scanned surface, as shown in Figure 6b. The lateral and height resolution of the laser scanner are 50 and 20 μm, respectively.

For each pavement surface, an area of 150 × 150 mm^2^ was selected to scan, which included at least 3000 × 3000 data points. The original point cloud data of each surface included noise points, discrete points, and some points outside the scanning area, as shown in Figure 6c. Therefore, the point cloud processing software Geomagic Wrap 2021 was used to edit the scanned point cloud of pavement surface, and a reduced area of 100 × 100 mm^2^ was cropped out (Figure 6d) for further study. Moreover, A series of post-processing was conducted on the original point data, including filling the missing data pixels, removing slope, and offsetting the base plane to the average plane (i.e., bringing the average height to zero). For a pavement type, 8 parallel samples were captured.

To understand the amplitude and wavelength of the scanned surface topography, the surface height distribution was plotted based on the probability density function of the height values, and the texture characterization of the four mixtures was investigated. Many studies have shown that regional statistical parameters are closely related to skid resistance [31,32,33]. To analyze the surface characterization, four parameters about height distribution, including root mean square height ($S_{q}$), skewness ($S_{sk}$), kurtosis ($S_{ku}$), and estimated mean texture depth (EMTP) will be considered respectively and calculated [34].

$S_{q}$ is equivalent to the standard deviation of heights within the defined area, representing the root mean square of ordinate values in the area, and is calculated by Equation (9).

(9) $$S_{q}=\sqrt{\frac{1}{A}\iint z^{2}\left(x,y\right)dxdy}$$

$S_{sk}$ is the quotient of the mean cube value of the ordinate values and the cube of $S_{q}$ within the defined area. It measures the asymmetry of the profile amplitude distribution from the baseline, which is affected by discrete peaks or valleys. When $S_{sk}=0$, it indicates that the texture is symmetrically distributed with the mean plane. When $S_{sk}<0$, it indicates that the valleys are sharp and narrow, while the peaks are smooth and wide. When $S_{sk}>0$, it indicates that the valley widths are flat and the peaks are sharp and narrow. The $S_{sk}$ can distinguish well the shape of surface texture. The mathematical expression is shown in Equation (10).

(10) $$S_{sk}=\frac{1}{{S_{q}}^{3}}\left[\frac{1}{A}\iint z^{3}\left(x,y\right)dxdy\right]$$

$S_{ku}$ represents the mean quartic value of the ordinate values and the fourth power of $S_{q}$ within the defined area, similarly to $S_{sk}$. When the surface texture distribution meets the normal distribution, $S_{ku}=3$. When $S_{ku}>3$, it indicates that the surface amplitude distribution is steep, which is called a sharp kurtosis surface. When the $S_{ku}<3$, the surface is called a low kurtosis surface with a relatively flat distribution. The mathematical expression is shown in Equation (11).

(11) $$S_{ku}=\frac{1}{{S_{q}}^{4}}\left[\frac{1}{A}\iint z^{4}\left(x,y\right)dxdy\right]$$

EMTD is the ratio between enclosed volume and the surface area. The enclosed volume is the volume that is covered by a space plane on the pavement surface.

#### 3.2.2. Dynamic Friction Test

**Figure 7:**
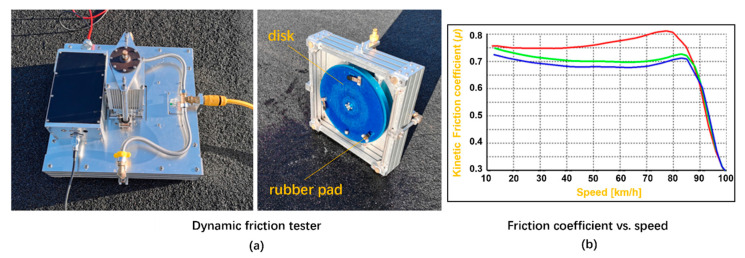
Dynamic friction tester (**a**) and results of friction coefficient curves (**b**).

The LHMC-0968 dynamic friction tester (DFT) was used to measure the COF of the four pavement surfaces in the field, as shown in Figure 7. The results of the DFT were used to verify the friction coefficient calculation based on the top topography.

The shore hardness of the rubber pads of DFT is about 58. To measure the dynamic friction of the selected surface, a DC electric motor in the DFT drives the disk to reach a rotation speed. Once the target speed of the rubber pads was obtained, the DC electric motor was switched off, and the disk with the rubber pads was then attached to the surface with a vertical contact pressure of 150 kPa. Due to the friction generated from the rubber pads and the surface, the speed of the pads was reduced to a full stop. At the braking phase, the effective COFs recorded ranged from 80 km/h to 0 km/h. For each pavement type, at least 8 parallel results of COF were captured.

#### 3.2.3. Modulus of Rubber

**Figure 8:**
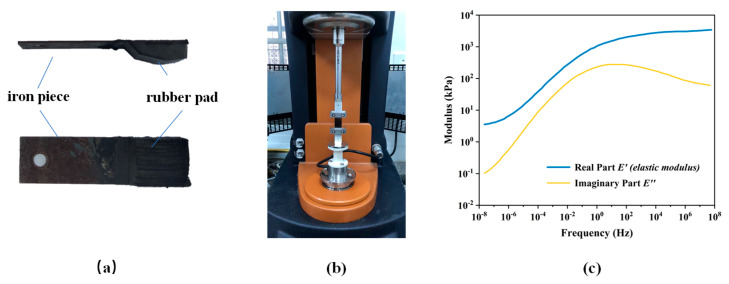
The rubber pad of the DFT (**a**) and frequency sweeping (oscillation) test to it (**b**) for the complex modulus (**c**).

The modulus of rubber is a key parameter for the friction estimation (recall Equation (2)), where the complex modulus is involved in calculating the theoretical friction coefficients (Equation (2)) and the elastic modulus is used to define the interfacial separation (Equation (8)). In this study, the modulus of rubber was obtained by the frequency sweeping (oscillation) test in the dynamic mechanical analysis (DMA) [35,36] on the rubber pad taken from the DFT, as shown in Figure 8a. The frequency sweeps ranged from 0.10–10 Hz with the strain level control at 0.8%, and the sweeping temperature ranged from −20 to 180 °C. Eventually, the master-curve at a reference temperature 20 °C (Figure 8b) was obtained by shifting and splicing the curves at each test temperature according to the time-temperature equivalence principle.

Although the sensitivity of rubber modulus to temperature, which would affect the friction in real time due to its viscoelasticity, the fixed modulus of rubber at a reference temperature of 20 °C was used for the derivation of the coefficient of friction and interfacial separation in this study due to the complexity of the inter-coupling of temperature, friction, and modulus. In other words, the effect of flash temperature on friction is not considered in the study.

## 4. Discussions and Results

### 4.1. Texture Characterization

**Figure 9:**
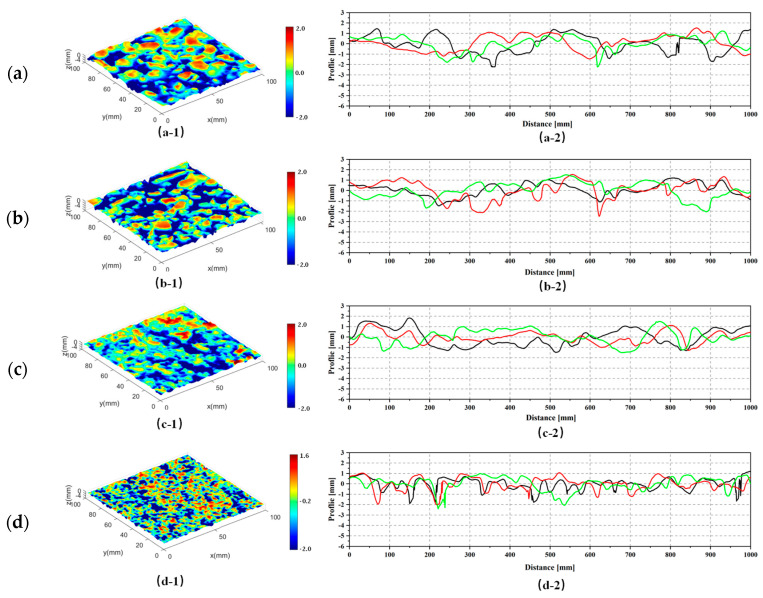
Top topographies and profiles of (**a**) SMA-16, (**b**) SMA-13, (**c**) AC-13, and (**d**) OGFC-5.

The total topographies of the four pavements were collected by the portable laser scanning, as shown in Figure 9(a-1–d-1). Their corresponding profiles (the red, black, and green lines represent three at three different cross sections) for each pavement surface were plotted as shown in Figure 9(a-2–d-2). For each pavement, the profiles show a similar pattern in heights and wavelengths. For different NMAS, the profiles of SMA-16 have higher heights compared to those of AC-13 and SMA-13, which indicates that a higher NMAS would create a rougher surface. In addition, the SMA-13 has a much rougher surface compared to the mixture AC-13, which has deeper valleys and higher peaks. The surface roughness size of OGFC-5 is smaller than that of others. On its surface, the heights of downward valleys are significantly greater than those of upward peaks, which was caused by a stronger compaction effect due to its intermittent gradation and minimal NMAS.

**Figure 10:**
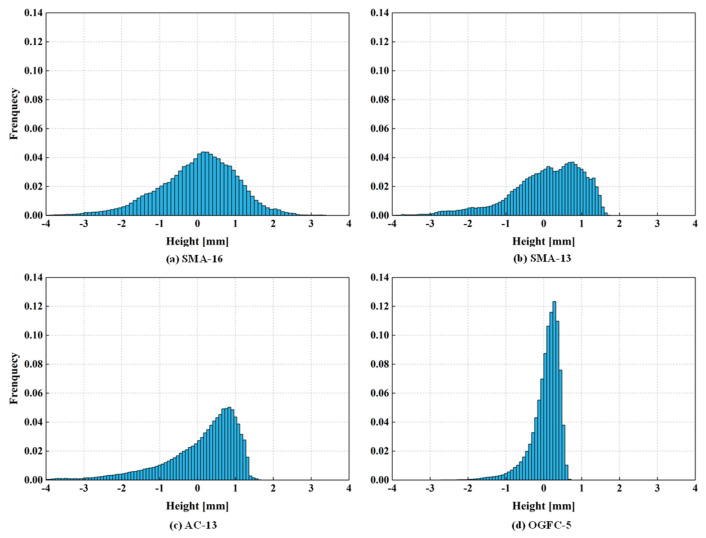
Height distributions of (**a**) SMA-16, (**b**) SMA-13, (**c**) AC-13, and (**d**) OGFC-5.

Surface height distributions (probability density function of the height values) of the four pavement surfaces are shown in Figure 10. In all distributions, the left tails are longer than the right tails, especially for OGFC-5, SMA-13, and AC-13. In other words, their height distributions are negative-skewed. The scanning work for all the pavements was conducted before they were first put into use. Therefore, it confirmed that compaction caused skewing of the surface topography as described above. For the OGFC-5 gradation, the peaks are sharp and narrow, which further indicates that most of the height of its top topography is within a narrow interval due to the compaction. In comparison, the height distribution of SMA-16 is closer to a symmetrical normal distribution, which seems to indicate that grade types with larger NMAS are less affected by the skewing effect due to compaction.

To quantitatively evaluate the heights and profile shapes of these pavement surfaces, the height distribution parameters were calculated based on the digitized pavement texture, as shown in Table 2. The MPD are 1.24 mm, 0.91 mm, 0.83 mm, and 0.53 mm, for SMA-16, SMA-13, AC-13, and OGFC-5, respectively. The results of the EMTD show that the average height was determined by the NMAS and gradation type. The mixture with gap-graded gradation shows a higher average height than that of the mixture with dense-graded gradation at the same NMAS, such as the SMA-13 and AC-13. All the $S_{sk}$ are smaller than 0, and the negative skewnesses of the four surfaces are ranked from highest to lowest as OGFC-5, AC-13, SMA-13, and SMA-16. A higher skewness of the topography indicates that there are more downward valleys on its surface, and those valleys are gentle and wide. The $S_{ku}$ are 3.629, 4.835, 6.331, and 8.019 for SMA-16, AC-13, SMA-13, and OGFC-5, respectively. The $S_{ku}$ of SMA-16 is around 3, which means its height distribution is close to a normal distribution, as shown in Figure 10a. However, the $S_{ku}$ of OGFC-5 is higher than 8. This indicated that a large amount of topography is concentrated at the same height.

### 4.2. Top Topography and the Cutting Depth

Depths of the cutting planes for the top topography of four pavements were calculated by the four methods described above, as listed in Table 3. It should be noted here that the base plane for calculating the depth of the cutting plane is not the one that crosses the highest point of the topography in the scanning area, but the one below the first 0.1% of data points, in order to avoid bias caused by individual isolated or singular points.

In the calculation of interfacial separation (u) (recall Equation (8)), the parameter $S_{q}$ is taken from Table 1. The roll-off wavevector ($q_{0}$) is approximately equal to 2π⁄NMAS, thus $q_{0}$ of SMA-16, SMA-13, AC-13, and OGFC-5 are 392, 483, 483 and 1256 $m^{-1}$, respectively. p is the vertical pressure on the rubber, 150 kPa. Since the effective test speed (v) of DFT is in the range of 20~80 km/h (≈5.5~22.2 m/s), the elastic modulus of the rubber ($E^{\prime }$) is taken from the DMA results (Figure 8c) with perturbing frequencies $\omega =q_{0}v$. Consequently, the u is not a fixed value in the speed range of 20~80 km/h.

**Figure 11:**
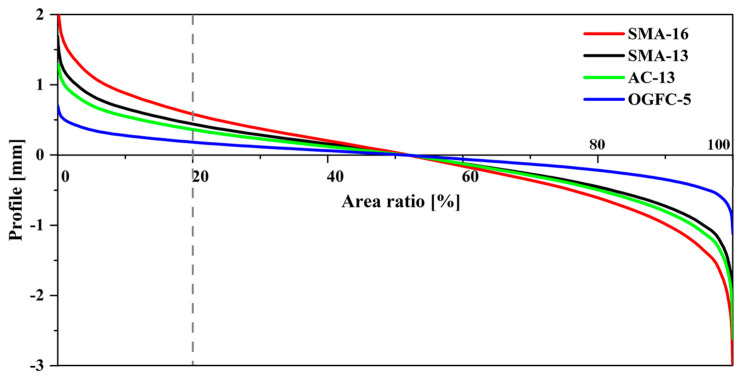
Transforming area ratio to depth for cutting plane using a bearing area curve (BAC).

When the fixed area ratio 20% of topography is used to define the cutting plane, it cannot be directly compared with the results of depth reported by the other three methods. To deal with it, the bearing area curve (BAC) of the surface topography was used to visualize the relation between area ratio and depth, as shown in Figure 11. BAC is introduced by Ech et al. [37] to evaluate the evolution of the surface texture during wear. In fact, BAC is a variant of the cumulative probability density function of the surface height; thus, the main parameters affecting its shape remain the mean, the standard deviation of the height distribution, and the skewness.

The area ratios of all cutting planes can be derived by the BAC based on the information of depths, as listed in the right part of Table 2. For SMA-16 with the lowest negative skewness, the area ratios of its top topography obtained by the four methods range from about 7% to no more than 50%. However, for the OGFC-5, which has the most negative skewness, the area ratios obtained by 0.5 $S_{q}$ and interfacial separation both indicated about 20%. Those indicate that the skewness information of the surface height distribution was neglected when the mean plane and the fixed area ratio method are used to describe the cutting plane.

**Figure 12:**
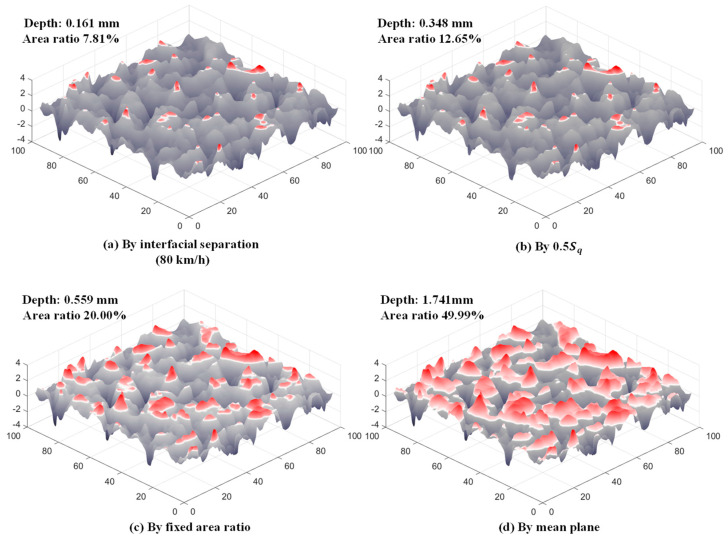
Visualization of the top topography of SMA-16 pavement (red marked) determined using (**a**) interfacial separation, (**b**) 0.5 $S_{q}$, (**c**) fixed area ratio, and (**d**) mean plane.

**Figure 13:**
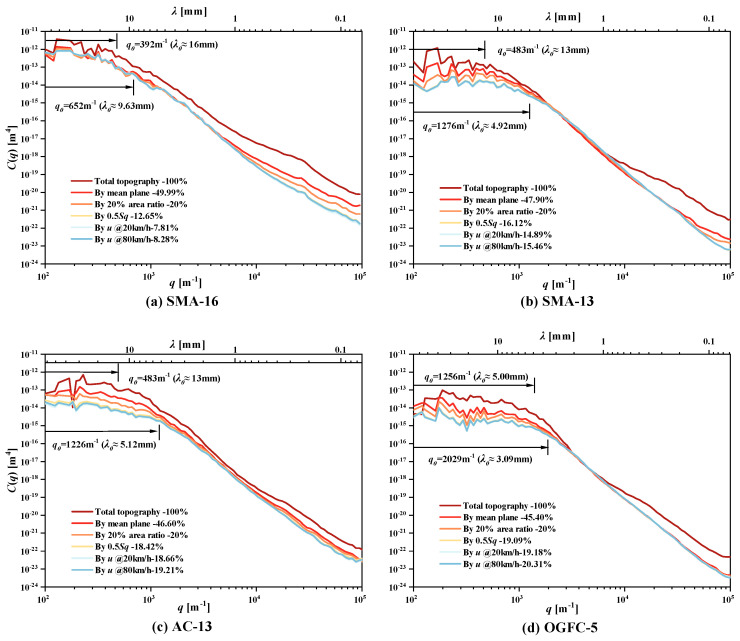
Top topographic PSD curves of (**a**) SMA-16, (**b**) SMA-13, (**c**) AC-13, and (**d**) OGFC-5.

Visualization of the top topography of the SMA-16 pavement (red marked) obtained by the four cutting methods is shown in Figure 12. Only a few tips of peaks were included in the top topography described by the interfacial separation, while almost all tips of peaks were included in that by the 0.5 $S_{q}$, and all peaks that rose out of the mean plane were included in the mean plane. We further calculated the PSD of the top topographies of the four pavements, which are obtained by four cutting methods and the 100% topography, as shown in Figure 13. The program used here refers to the open-source MATLAB 2022b script [38] shared by Mahboob Kanaf. Since the effective lateral resolution of the laser scanner is 50 um, wave vectors with wavelengths less than this value were discarded in calculating these PSD curves. For each pavement, the thinner top topography was used to calculate C(q), and the shorter wavevector was accounted for in the wave vector clusters. In other words, much of the long-scale roughness and part of the short-scale roughness that constituted the total texture of the pavement surface were filtered out, especially for those with wavelengths longer than or equal to NMAS. Therefore, at thinner top topographies, the values of C(q) corresponding to a *q* are relatively small, especially when the wavelength of q is longer than $q_{0}$. In addition, the $q_{0}$, which Persson used as the dividing point between the gentle and inclined segments of the C(q) curve, also corresponds to a smaller value in thinner topography. This implies that if the depth of the penetration of the rubber into the pavement surface asperities is at this deep level, the rubber cannot see the longer wavelengths that exist beyond this level in practice.

### 4.3. Kinetic Coefficient of Friction: Experimental vs. Theoretical

**Figure 14:**
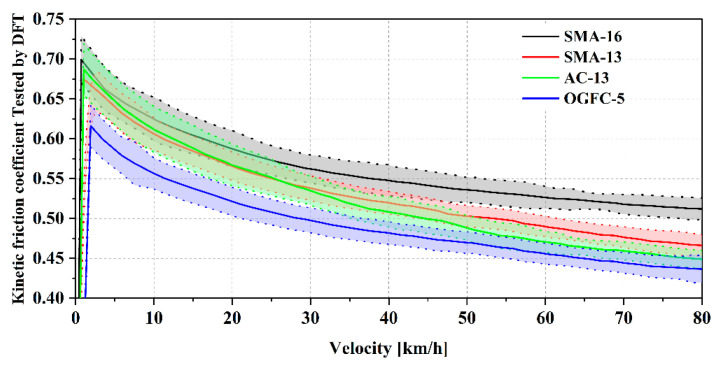
Kinetic coefficient of friction of the four pavements measured using DFT.

The results of the DFT in the field are shown in Figure 14. The measured pavement temperatures were about 19.5 °C for SMA13 and 20.6 °C for the other three. Therefore, no temperature correction was performed on the data, due to the close temperature. Moreover, the subsequent calculation of the theoretical friction coefficient was performed at a reference temperature of 20 °C. Every curve represents an average of 8 repeated measurements. The shading of each curve represents the standard deviation (error) of those repeated results. Friction coefficients of all four pavements decreased with increasing speed. At a lower speed, 10 km/h, the ranking of the friction coefficients from large to small is SMA-13 > AC13 > SMA13 > OGFC-5, but this ranking is almost inverted at 80 km/h, which is SMA16 > SMA13 > AC13 > OGFC-5.

**Figure 15:**
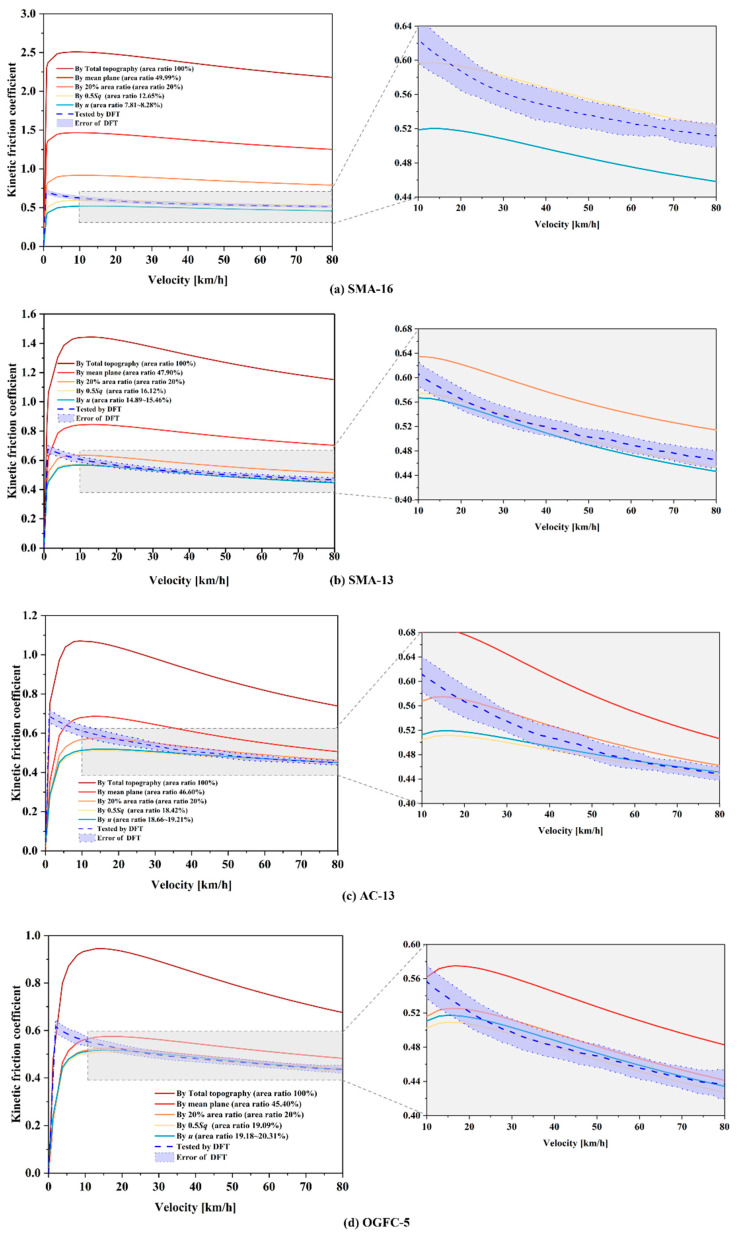
Comparison of COF results obtained by estimation of top topography PSD and field experiment for (**a**) SMA-16, (**b**) SMA-13, (**c**) AC-13, and (**d**) OGFC-5.

The estimated COFs of the four pavements are shown in Figure 15, which were estimated based on the top topographic PSD (Figure 13) and Persson’s rubber friction theory (Equations (2)–(4) and (6)).

Each curve represents an average of 8 repeated calculations. For the four pavements, the estimated COF first increases and then decreases as the speed increases, and the greatest values were obtained in about 0~10 km/h. The blue dashed lines and their surrounding shading represent the DFT results and the corresponding errors. The theoretical COFs derived from 100% and 50% topography are much higher than the measured values and other calculated results. It verifies Mahboob Kanafi’s statement that even though the correct scaling and short-scale roughness components are well preserved in the calculated top topography PSD, the calculations of the contact mechanics using any top topography PSD other than the top 50% lead to false conclusions [25]. Therefore, from the four subplots on the left side of Figure 15, it can be concluded that the schemes for estimating the COF by 100% and 50% of the top texture are not feasible. Therefore, we further focus on comparing the measured values with the estimated values calculated by 0.5 $S_{q}$, 20% top topography and interfacial separation, as shown in the local enlargement on the right side of Figure 15.

The trends of the measured and the estimated values of COF with the increasing speed are approximately identical when speeds > 20 km/h, but significantly different when speeds ≤ 20 km/h. The measured COF decreases monotonically with increasing velocity, and the slope increases closer to the zero point. However, there is a peak in the curve of COF estimates, which is in the range of 10 to 20 km/h. On the left side of this peak, COF increases sharply with increasing speed, while on the right side of the peak, it decreases monotonically at a gradually slowing rate. This difference should be attributed to adhesion, which is one of the main components of friction, and it cannot be neglected at lower velocities. However, it was actually neglected in the calculation of the theoretical COF at all velocities.

For SMA16, the measured COF curve is between the two calculated COF curves derived from the top topographies determined by the 0.5 $S_{q}$ (area ratio 12.65%) and interfacial separation (area ratio 7.81~8.26%), respectively. For SMA-13, the measured curve is slightly above the calculated curves determined by the 0.5 $S_{q}$ (area ratio 16.12%) and the interfacial separation (area ratio 14.89~15.46%). For the surfaces AC-13 and OGFC-5, the estimated curves of the 0.5 $S_{q}$, the interfacial separation, and the 20% area ratio are relatively close, and the measured curves are also close to the three estimated curves, especially close to the curves of 0.5 $S_{q}$. One conclusion that can be drawn is that when estimating COF curves for pavements with large negative skewness, the results obtained using the top topography with larger area ratios are closer to the measured results.

**Figure 16:**
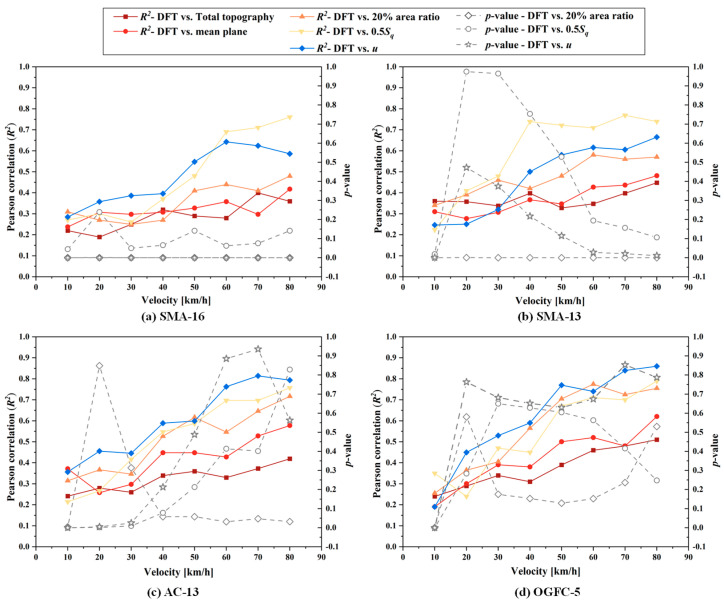
Pearson correlation and *p*-value between the estimated COF and measured COF for (**a**) SMA-16, (**b**) SMA-13, (**c**) AC-13, and (**d**) OGFC-5.

We further conducted significance tests for the measured COF curve and the three closest estimated COF curves (by the 0.5 $S_{q}$, the interfacial separation and the 20% area ratio) for each pavement. At each velocity point, the COF was measured and estimated eight times. The parameter of significance level α was set as 0.05. If the calculated p-value is greater than 0.05, it indicates that there is no significant difference (a confidence level of 95%) between the COF results by the DFT and the estimated method. Results of the significance test are shown on the right axis of Figure 16. For all four pavements, the p-value between the measured curve and the 0.5 $S_{q}$ estimated curve were greater than 0.05 at velocities ≥ 40 km/h. This indicates that there is no statistical difference between this estimated mean COF and the measured mean COF at high speeds. The estimated COF by interfacial separation successfully predicted the friction for the AC-13 and OGFC-5 (the p-value ≥ 0.05 at most velocity points) but failed for SMA-16 and SMA-13 (the p-value < 0.05 at most velocity points). However, the estimated COF by a fixed 20% area ratio only successfully predicted the friction for the OGFC-5. Those indicate that only 0.5 $S_{q}$ provides a more reliable prediction of COF regardless of the skewness of pavements. Moreover, the predictions given by the interfacial separation are smaller on pavements with less skewness, while the predictions given by the fixed 20% area ratio are significantly larger due to the fact that neither of them carries information on the skewness of the texture.

In addition to the significance test for the mean estimated COF and mean measured COF, we also checked the Pearson correlation with the measured COF s of the five types of estimated values at velocities of 10, 20, 30, 40, 50, 60, 70, and 80 km/h, respectively, as shown in left axis of Figure 16. For each pavement, the highest correlations with the measured data were found in the estimated one, the mean of which is the closest to the mean of the measured data. The estimated COFs obtained by the half and total topography also have slight correlations (*R*^2^ ≤ 0.5) with the measured COFs, although there are significant gaps between their mean values. The correlations at high velocities are larger than those at low velocities for all the estimated COFs and measured COFs. The highest correlation is 0.79 for SMA-16 obtained with 0.5 $S_{q}$ at 70 km/h, 0.76 for SMA-13 obtained with 0.5 $S_{q}$ at 70 km/h, 0.81 for AC-13 obtained with interfacial separation at 70 km/h, and 0.85 for OGFC-5 obtained with interfacial separation at 80 km/h. For four pavements, at lower velocities (≤30 km/h), almost no correlation coefficient between predicted data and measured data exceeded 0.5, although the extreme values of *p*-values all occur in this velocity interval. This is due to the fact that the measured and predicted COF curves cross in the range of low velocities, causing the mean of their data to be similar, but for an individual within the data set, the measured and predicted values are not coordinated. However, in the high-speed interval, the measured COF and predicted COF values for an individual is gradually harmonized, although a slight gap is developed between their mean values of measured and predicted values. Those indicate that the COF of the pavement surface can be predicted more reliably and accurately by top topography PSD than by full topographic PSD, where the top topographic method using 0.5 $S_{q}$ cut planes is more applicable for more pavements and less affected by the skewness, while the method by interface separation is only suitable for pavements with large texture skewness.

**Figure 17:**
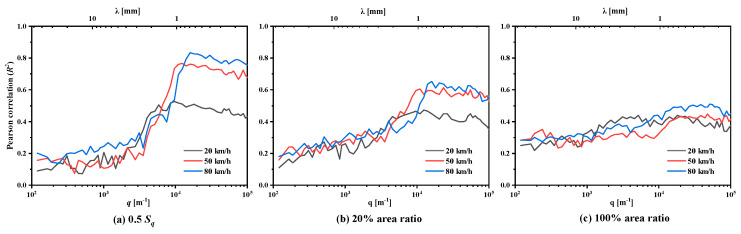
Correlation between all the COF measurements and all the top topography surface roughness PSD at all length scales for (**a**) 0.5 $S_{q}$, (**b**) 20% area ratio, and (**c**) 100% area ratio.

In order to see the robustness and stability of the COF estimation approach using 0.5 $S_{q}$-top topography PSD, we further investigated the frequency-wise correlation between all the COF measurements and all the top topography surface roughness PSD (i.e., measured COF vs. C(q) at each λ). The results are shown in Figure 17a, and it is compared with the results of a 20% area ratio (Figure 17b) and a 100% area ratio (Figure 17c). The results of λ < 50 μm are avoided in Figure 17 because of the inaccuracies of the optical profiler at these high frequencies (short wavelengths). It should be noted here that each correlation curve was the analysis result of 32 pavement samples (4 pavement types × 8 parallel samples). There are two main facts that can be deduced from Figure 17.

First, the shorter wavelengths (λ<1 mm) contribute the most to rubber friction, especially those on the top topography. In comparison, the 0.5 $S_{q}$ cutting plane, as far as possible, make those shorter wavelengths that are actually involved in the rubber contact be used to estimate the COF. When using 0.5 $S_{q}$ as the cutting plane of the top topography, a high correlation, *R*^2^ > 0.8, was observed between the roughness below 1 mm wavelength and the friction at speeds of 50 km/h and 80 km/h (the red and blue lines in Figure 17a). However, the correlation respectively decreased to about 0.6 and below 0.5 when the top 20% and 100% top topography was used to estimate the COF (the red and blue lines in Figure 17a,b). This is because the deeper cutting plane causes some roughness with short wavelengths at the bottom of the valley that does not participate in contact with the rubber to be accounted for in the estimated friction.

Second, the top topography PSD cannot be used to estimate the friction at lower speeds. Although the shorter wavelengths on the 0.5 $S_{q}$ contribute the most to rubber friction at 50 km/h and 80 km/h, this is only in terms of the hysteresis of friction. At 20 km/h, the highest correlation decreased to about 0.5 (the black line in Figure 17a), which indicates that those shorter wavelengths are not the only contribution to rubber frictions.

It should be noticed here that those longer wavelengths (λ>1 mm) on the 0.5 $S_{q}$-top topography seems to be less correlated with the friction. But it cannot be concluded that those long wavelengths do not contribute to the friction because they are incomplete waves generated by intercepting the 0.5 $S_{q}$ plane after truncation. And the original long-waves (λ>1 mm) actually still have some correlation with the friction, the $R^{2}$ in the range of 0.3~0.4 as shown in Figure 17c.

## 5. Summary and Conclusions

This study describes a method of estimating kinetic COF for asphalt pavements using the top topography PSD. It is an improvement on Persson’s friction coefficient calculation method, which uses the top topography PSD instead of the total topography PSD to estimate the hysteresis excitation energy dissipation of rubber. The purpose of this is to avoid some roughness at the bottom of the skewed pavement profile being involved in the calculation of friction, but which does not actually make real contact with the rubber.

The core of the method is the selection of the cutting plane of top topographies. We compared the effects of four cutting methods for top topographic regions on friction prediction results based on four typical asphalt pavements with various skewness. The four methods include the 0.5 $S_{q}$ recommended by Hartikainen, the fixed area ratio (top 20%) by Mahboob, the interface separation, and the upper half of the top topography (top 50%) by Persson. It was found that when the fixed top 20% and top 50% topography are used, the mean estimation of COF was significantly larger than the mean measured value (p-value≪0.05), especially for pavements with small negative skewness, since these two cutting methods do not carry information on the deflections of the pavement. The interface separation method can predict pavements with large negative skew very well, but gives small estimates for pavements with low negative skew. 0.5 $S_{q}$ has the strongest versatility for pavements with various skewness, and there is no statistically significant difference between the mean estimate and mean measured values for all four pavements (p-value>0.05).

Among the four cutting methods, the 0.5 $S_{q}$, as far as possible, makes the shorter wavelengths (λ<1 mm) that are actually involved in the rubber contact were used to estimate the COF, so that high correlations (*R*^2^ > 0.8) are observed between the roughness below 1 mm wavelength and the friction. However, the correlations are only about 0.6 and below 0.5 when the top 20% and total top topography were used to estimate the COF. It should be noted that the top topography PSD can be used to estimate the COF only when the rubber sliding velocity is greater than about 30 km/h, since the hysteresis is not the only contribution of rubber frictions at low velocities. Therefore, only low correlations ($R^{2}$ = 0.4~0.5) were observed between the shorter wavelengths (λ<1 mm) on the top 0.5 $S_{q}$ topography and measured COF at low velocities.

In general, using 0.5 $S_{q}$ as the cutting plane to obtain the top topography and its PSD, and then estimating the friction by Persson’s theory is a more relevant and accurate method as of now. However, as mentioned before, the approach derived from Persson’s theory ignores the effect caused by adhesion, which may cause errors in the friction estimation at low speeds.

## Figures and Tables

**Table 1:** Mixture design parameter of the four mixtures.

Pavement Type	Location and Type of Roads	Binder Content	Additive	Asphalt Type
SMA-16	Part of the main line of S21 in Xinjiang	4.9%	\	SBS modified
SMA-13	Main line of G4 in Henan	6.5%	0.055% fiber	SBS modified
AC-13	Collector road of G4 in Xinjiang	4.8%	\	70# asphalt
OGFC-5	Part of the main line of S21 in Xinjiang	5.5%	Polyester fiber	SBS modified

**Table 2:** Height and shape parameters of the eight pavement surfaces.

Surface	EMTD [mm]	$S_{q}$	$S_{sk}$	$S_{ku}$
SMA-16	1.24	0.696	−0.291	3.629
SMA-13	0.91	0.970	−0.940	4.835
AC-13	0.83	1.026	−1.294	6.331
OGFC-5	0.53	0.386	−1.829	8.019

**Table 3:** Depths of the cut planes for the top topography of the four pavements by four methods.

Surface	Depth of the Cut Plane [mm]				Area Ratio of the Cut Plane [%]			
	By Mean Plane	$\mathrm{By}\;0.5\;S_{q}$	By 20% Area Ratio	By Interfacial Separation (20~80 km/h)	By Mean Plane	$\mathrm{By}\;0.5\;S_{q}$	By 20% Area Ratio	By Interfacial Separation (20~80 km/h)
SMA-16	1.741	0.348	0.559	0.161~0.180	49.99	12.65	20.00	7.81~8.28
SMA-13	1.523	0.485	0.611	0.424~0.440	47.90	16.12	20.00	14.89~15.46
AC-13	1.407	0.513	0.562	0.521~0.538	46.60	18.42	20.00	18.66~19.21
OGFC-5	0.562	0.193	0.226	0.197~0.211	45.40	19.09	20.00	19.18~20.31

## Data Availability

The original contributions presented in this study are included in the article. Further inquiries can be directed to the corresponding author.
